# Pathogenesis and Therapeutic Strategies Related to Non-Alcoholic Fatty Liver Disease

**DOI:** 10.3390/ijms23147841

**Published:** 2022-07-16

**Authors:** Tieshan Teng, Shuai Qiu, Yiming Zhao, Siyuan Zhao, Dequan Sun, Lingzhu Hou, Yihang Li, Ke Zhou, Xixi Yu, Changyong Yang, Yanzhang Li

**Affiliations:** 1 Institute of Biomedical Informatics, School of Basic Medical Sciences, Henan University, Kaifeng 475004, China; tengtieshan@vip.henu.edu.cn (T.T.); qiushuaitongxue@163.com (S.Q.); 1922010228@henu.edu.cn (Y.Z.); 1922010114@henu.edu.cn (S.Z.); 1921010168@henu.edu.cn (D.S.); 2021010054@henu.edu.cn (L.H.); 1922010094@henu.edu.cn (Y.L.); 1922010137@henu.edu.cn (K.Z.); 1922010110@henu.edu.cn (X.Y.); 2 School of Nursing and Health, Henan University, Kaifeng 475004, China

**Keywords:** NAFLD, steatosis, inflammation, hepatic fibrosis

## Abstract

Non-alcoholic fatty liver disease (NAFLD), one of the most common types of chronic liver disease, is strongly correlated with obesity, insulin resistance, metabolic syndrome, and genetic components. The pathological progression of NAFLD, consisting of non-alcoholic fatty liver (NAFL), non-alcoholic steatohepatitis (NASH), and liver cirrhosis, is characterized by a broad spectrum of clinical phenotypes. Although patients with mild NAFL are considered to show no obvious clinical symptoms, patients with long-term NAFL may culminate in NASH and further liver fibrosis. Even though various drugs are able to improve NAFLD, there are no FDA-approved medications that directly treat NAFLD. In this paper, the pathogenesis of NAFLD, the potential therapeutic targets, and their underlying mechanisms of action were reviewed.

## 1. Introduction

**Figure 1:**
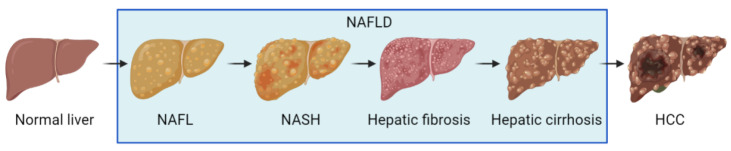
Schematic presentation of NAFLD progression.

Non-alcoholic fatty liver disease (NAFLD) is an umbrella term for a chronic liver disease caused by agents other than alcohol. It is characterized by excessive fat deposition, or steatosis, in the hepatocytes [1]. Pathological processes involved in NAFLD consist of non-alcoholic fatty liver (NAFL), characterized by harmless liver cell steatosis, non-alcoholic steatohepatitis (NASH), and variable degrees of fibrosis upon liver biopsy [2]. Clinically, liver cirrhosis and hepatocarcinoma are also attributed to NAFLD (Figure 1) [3]. Globally, the prevalence of NAFLD is about 25%, which threatens the health of adults, while generally harming children and adolescents [4,5]. The prevalence of NAFLD in Asia is about 29.62%, showing a continually increasing trend [6]. In the United States, more than one-third of the mortalities associated with liver diseases and diabetes were correlated to NAFLD [7]. It is common sense that the incidence of NAFLD is closely associated with the prevalence of liver cirrhosis and hepatocellular carcinoma [8].

Pathogenic factors for NAFLD are composed of eating habits, cardiovascular diseases, genetic polymorphisms of various genes, etc. [9]. During the development of NAFLD from liver steatosis, or liver inflammation to fibrosis, patients are exposed to multiple complications, such as hypertension, atherosclerosis, and other diseases [10,11,12]. However, the specific pathogenesis of NAFLD has not been elucidated, and chemotherapeutic options that target NAFLD have not been approved. Thus, a safe and effective treatment option for NAFLD is urgently needed. Based on recent reports, these potential action targets and therapeutic drugs mainly focus on metabolic disorders, steatosis, oxidative stress, inflammation, apoptosis, and fibrosis. Several therapeutic drugs with the potential for treating NAFLD have been characterized, and drug therapy strategies for targeted NAFLD treatment are likely to be implemented.

In the present paper, we aimed to summarize recent advances in the pathogenesis of NAFLD, review the bio-molecules identified as upcoming pharmacological targets for this disease, and highlight the most recent evidence for novel investigational agents, as well as currently marketed drugs.

## 2. The Pathogenesis of NAFLD

### 2.1. Two Hits Hypothesis 

Based on the previous report, the “two hits hypothesis” and “multiple hits hypothesis” were proposed to explain the pathogenesis of NAFLD. According to the “two hits” theory, imbalances in glucose and lipid metabolism lead to excessive accumulation of fatty acids [13]. As a critical toxic component in liver cells, fatty acids are able to elevate oxidative stress and inflammatory factors that induce hepatocyte damage, an essential pathological stage of NAFLD. Accordingly, the first “hit” is associated with lipid metabolism disorders, characterized by insulin resistance and the decrease in adiponectin, leptin, and other major adipocytokines. The second “hit” is highly correlated with steatosis, endoplasmic reticulum stress, oxidative stress, hepatocyte inflammation, and fibrosis. 

### 2.2. Multiple Hits Hypothesis

**Figure 2:**
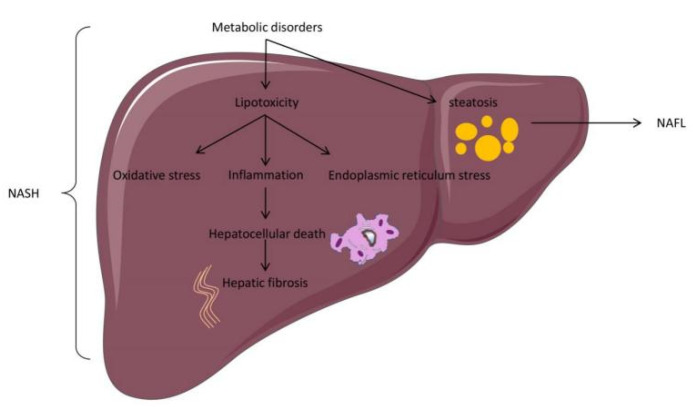
Basic pathological changes in an NAFLD model.

However, the causal relationship between the first “impact” and the second “impact” has not been determined [14]. The “multiple hits hypothesis” is considered to accurately explain NAFLD pathogenesis. According to this view, NAFLD pathogenesis is jointly induced by multiple factors, including insulin resistance, nutritional factors, oxidative stress, inflammatory factors, obesity, type 2 diabetes, hormones, gut microbiota, and epigenetic factors [15]. At present, insulin resistance and liver-free fatty acids (FFA) are considered to play an essential role in NAFLD pathogenesis [16]. In detail, glucose and lipid metabolism disorders can produce excess FFA, which enters the liver cells and transforms into triglycerides. The accumulation of triglycerides in the hepatocytes produces lipid droplets and triggers NAFL. The main pathological feature of NAFL is that liver cell steatosis exceeds 5%, which can be diagnosed by imaging. Furthermore, excess FFA enhances endoplasmic reticulum pressure, mitochondrial pressure, and the production of reactive oxygen species in the liver, leading to the production of inflammation, namely NASH. The pathological features of NASH include portal vein and lobular inflammation, as well as hepatocyte injury. With the development of NASH, some hepatocytes undergo apoptosis or necrosis and further produce inflammatory factors, which activate hepatic stellate cells to initiate liver fibrosis [17] (Figure 2). Liver fibrosis is the excessive expression and accumulation of extracellular matrix proteins in the liver, and it is an adverse consequence of hepatitis, which continues to develop into cirrhosis and liver cancer, requiring liver transplant for treatment [18,19]. Tackling NAFLD-associated fibrosis from different directions using combinatorial drug treatment and effective lifestyle changes holds the greatest prospect for success [20].

### 2.3. Progress on the Pathogenesis of NAFLD

In recent years, some new progress has been reported involving the pathogenesis of NAFLD, including pro-inflammatory diets, gut metabolome, adipose tissue inflammation, genetic factors, etc. [21]. Clinical studies provide evidence that pro-inflammatory diets enriched with fructose, trans-fatty acids, and trimethylamine N-oxide (TMAO), such as fried foods, refined grains, and red meat, are associated with an increased risk for progressing NAFLD [22]. In contrast, a low-sugar, carbohydrate-restricted isocaloric, or Mediterranean diet have been demonstrated to be beneficial to improve NAFLD. Moreover, considerable progress has been made for intestinal microbiota in patients with NAFLD in recent years. Recent studies have confirmed that Bacteroides abundance is increased and Prevotella abundance is decreased in patients with NASH. Advanced fibrosis was associated with an abundance of proteobacteria and *Escherichia coli* and a decrease in Firmicutes, such as *Faecalibacterium prausnitzii*. Experimental evidence also indicated that endotoxin-producing strains such as *Enterobacter cloacae* b29, *Escherichia coli* py102, and *Klebsiella pneumoniae* a7 promoted NAFLD in germ-free mice on a high-fat diet [23]. Furthermore, bacterial metabolite screens were used to investigate the metabonomic characteristics of NAFLD patients. Numerous studies have demonstrated that imidazole propionate, n,n,n-trimethyl-5-aminovaleric acid, and 3-(4-hydroxphenyl) lactate are increased in the serum of patients with NAFLD [24,25]. Specifically, 3-(4-hydroxphenyl) lactate can be used to discriminate NAFLD subjects, with and without fibrosis. Lastly, the increased expression of several chemokines, including c-c motif chemokine ligand 2 (ccl2), ccl5, and ccl13, were able to recruit at the macrophages (ATM) and normal T cells and to initiate at inflammation in obese patients. Tumor necrosis factor alpha (TFNα), interleukin 1-beta (il-1β), and il-6 were demonstrated to be adipokine associated with insulin resistance, at inflammation, and fatty liver, and their expression was also increased in human obesity [26]. Gut inflammation is able to increase systemic inflammation, and deteriorate liver disease and insulin resistance, exemplifying aspects of a gut–liver axis.

## 3. Potential Targets for NAFLD Treatment 

**Figure 3:**
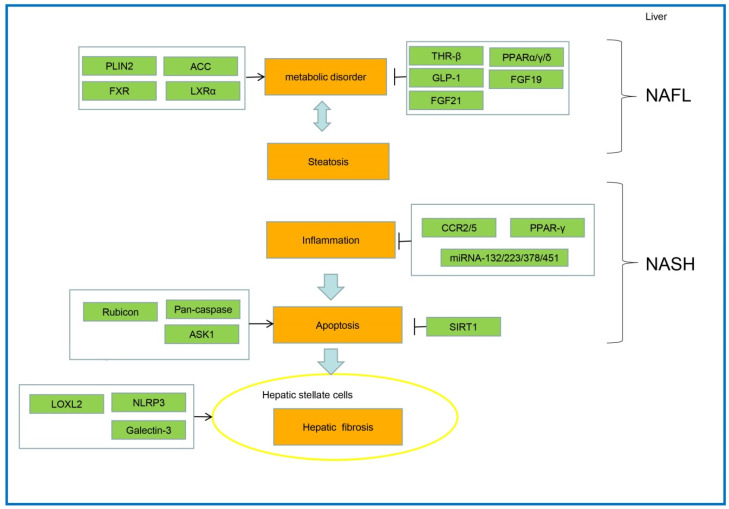
The pathogenesis and key therapeutic targets for NAFLD.

Several critical factors involved in NAFLD progression were reported as potential therapeutic targets. These crucial factors alleviate NAFLD, mainly by inhibiting metabolic disorders, steatosis, oxidative stress, inflammation, apoptosis, and liver fibrosis (Figure 3).

### 3.1. Potential Targets of Metabolic Disorders/Steatosis in NAFL 

#### 3.1.1. Sodium-Glucose Cotransporter 2 (SGLT2)

SGLT2 is mainly distributed in the proximal tubular curvature and is responsible for the re-absorption of 80–90% of glucose in the kidney. SGLT-2 inhibitors are glucose-lowering agents that improve glucose control, while promoting weight loss and lowering serum uric acid levels [27]. In clinical trials for the treatment of NAFLD patients, several SGLT2 inhibitors, such as canagliflozin and dapagliflozin, were able to prevent lipid accumulation in the liver and decrease the levels of liver transaminase through promoting urinary glucose excretion [28]. NGI001, a novel selective inhibitor of SGLT2, was used to treat high-fat diet (HFD)-induced nutritive obesity mice [29]. The results exhibited that NGI001 could inhibit the secretion of fat factors associated with insulin resistance and liver lipid accumulation. Empagliflozin, another inhibitor of SGLT2, was found to enhance fat consumption and reduce insulin resistance in obese mice induced by HFD [30]. In recent years, the available evidence has also shown that the other SGLT-2 inhibitors, consisting of ipragliflozin, tofogliflozin and luseogliflozin, can attenuate endoplasmic reticulum (ER) stress, oxidative stress, low-grade inflammation, autophagy, and apoptosis in NAFLD patients [31]. Given this evidence, SGLT-2 inhibitors are emerging as promising agents.

#### 3.1.2. Perilipin Drip Protein-2 (PLIN2)

PLIN2 is a lipid droplet (LD) coat protein which is the most abundant member of the PLIN protein family in the steatotic livers of humans and mice. The expression of PLIN2 is strongly correlated with the severity of steatosis. Overexpression of Plin2 increases triglyceride (TAG) accumulation and reduces lipolysis by excluding the adipose triglyceride lipase (ATGL), which is located on the surface of LD. In addition to regulation of lipolysis, PLIN2 has also been implicated in the regulation of hepatic lipophagy, with increased rates of autophagy observed in livers of whole-body deficient PLIN2 mice. Moreover, it was reported that the levels of cholesterol and triglycerides in the livers of mice with whole-body deletion of PLIN2 were significantly reduced when fed on a Western diet for 30 weeks (*p* < 0.001) [32]. Furthermore, several studies have shown that PLIN2 deletion can lead to the production of a large number of ω-3 and ω-6 long-chain fatty acids (FA) in mouse hepatocytes, which will reduce cholesterol synthesis and lipogenesis (*p* < 0.05) [32]. Histological observation also verified that the degree of steatosis in PLIN2-deficient mice was much lower than in wild-type mice [33]. These results suggested that PLIN2 could be taken as a potential therapeutic target to alleviate steatosis, although more trials need to be performed to verify its function in the human body.

#### 3.1.3. Liver X Receptor α (LXRα)

LXRα is a vital regulator of glucose homeostasis and insulin sensitivity [34]. LXR activation is considered promising for modulating cholesterol homeostasis, inducing anti-inflammatory effects and increasing insulin sensitivity (*p* < 0.001). LXRα activation also induces de novo lipogenesis (DNL) and decreases LDL catabolism, alleviating hepatic steatosis [35]. Treatment with an LXRα agonist, SR9243, remarkably decreased the severity of hepatic inflammation and reduced hepatic fibrosis in NASH mice [36]. Oltipraz, a synthetic 1, 3-dithiole-2-thione with an antisteatotic effect by inhibiting LXRα, is capable of significantly decreasing the liver fat content in NAFLD patients [37]. These findings exhibited that LXR agonists could be therapeutically crucial in NASH treatment.

#### 3.1.4. Acetyl CoA Carboxylase (ACC)

ACC is a biotin enzyme in the first stage of fatty acid synthesis, and it is able to catalyze the ATP-dependent condensation of carbonate and acetyl-CoA to form malonyl-CoA, which plays an essential role in fatty acid metabolism. In mammals, the ACC enzyme possesses two isoforms consisting of ACC1 and ACC2. The expression of ACC1 occurs primarily in lipogenic tissues, such as liver, adipose, etc. ACC2 expression occurs primarily in oxidative tissues, including heart, skeletal muscle, etc. ACC1 is located in the cytosol and accounts for the rate-control in de novo lipogenesis (DNL). In contrast, ACC2 is located in the mitochondrial membrane and negatively regulates fatty acid oxidation by producing localized malonyl-CoA, which allosterically inhibits carnitine palmitoyltransferase I (CPT1) and the transfer of long-chain CoAs into the mitochondria.

Previous studies indicated that reducing the hepatic expression of ACC1 and ACC2 by antisense oligonucleotides (ASOs) leads to significant reductions in hepatic triglyceride content and the reversal of hepatic insulin resistance in high-fat diet-induced NAFLD mice (*p* < 0.001). In addition, ACC1/ACC2 inhibitor, ND-630, was used to treat obese rats induced by HFD. The results suggested that the steatosis of the rat liver was inhibited, and the insulin sensitivity was improved significantly [38]. GS-0976 (febuxostat), another inhibitor ofACC1 and ACC2, could reduce liver fat by 29% when used to treat patients with NASH and fibrosis. Furthermore, it was found that the median value of DNL decreased by 22%, compared to the baseline, after GS-0976 treatment [39,40].

#### 3.1.5. Fatty Acid Synthase (FASN)

Fatty acid synthase (FASN) is one of the most promising targets in the treatment of NAFLD because it is the most critical rate-limiting enzyme of hepatic DNL. FASN is highly expressed in patients with NAFLD, as well as in several types of cancer associated with dysregulated lipid metabolism, such as colorectal cancer and HCC. FT-4101, a potent and selective FASN inhibitor, has been shown to prevent hepatic steatosis, inflammation, and fibrosis in rodent models of NAFLD [41]. Previous clinical trials have demonstrated that an FASN inhibitor, called TVB-2640, is able to inhibit hepatic DNL, reduce liver fat, and improve hepatic steatosis without significant safety concerns in patients with NAFLD (Number NCT03938246) [42]. With hepatocyte-specific FASN knockout, hypoglycemia and fatty liver development was detected in mice during zero-fat feeding or after fasting. In addition, TVB-2640 can reduce liver fibrosis by virtue of the critical role of FASN in the activation of fibrogenesis in hepatic stellate cells [43]. MicroRNA-103 is also reported to be capable of repressing DNL and dampening the development of HFD-induced NAFLD through targeting at FASN and Scd1 in the mouse liver [44]. Thus, FASN is one of the most attractive targets for NAFLD treatment.

#### 3.1.6. AMP-Activated Protein Kinase (AMPK)

AMPK is a highly conserved central regulator of hepatic lipid and glucose metabolism, whose activation is considered to be therapeutically beneficial for the treatment of NASH [45]. Once activated, AMPK can prevent fatty acid and cholesterol synthesis through inhibiting the phosphorylation of HMGCR and SREBP1C. Liver-specific AMPK activation can also decrease the expression of inflammation and fibrosis genes, thus reducing triglyceride accumulation while increasing CPT1 expression in hepatocytes. Similarly, Mice expressing an AMPK mutation in the liver display lower rates of lipogenesis and protection from NAFLD and insulin resistance when fed a high-fructose diet. Boudaba et al. have shown that a thienopyridine family of AMPK activators (A-769662) targeting the liver can improve liver steatosis and glucose parameters in obese mice [46]. Several studies have shown that a range of natural products, consisting of berberine, resveratrol, demethyleneberberine, nicotine, etc., are potential therapeutic agents for NAFLD via the activation of the AMPK signaling [47,48,49]. These findings indicate that AMPK is a promising target for the treatment of NAFLD.

### 3.2. Potential Targets of Hepatic Inflammation in NASH

#### 3.2.1. Apoptosis Signal-Regulated Kinase 1 (ASK1)

Apoptosis signal-regulated kinase 1 (ASK1), a member of the mitogen-activated protein kinase (MAP3Ks) family, is an upstream activator of the c-Jun N-terminal kinase 1 (JNK1) and p38 MAPK signaling cascades. ASK1 can be activated by various stressors, such as intracellular TNF-α, endoplasmic reticulum stress, and reactive oxygen species (ROS), leading to subsequent induction of the activation of JNK and p38 MAPK. Activated JNK and p38 MAPK regulate autophagy, thereby modulating apoptosis, inflammation, and fibrosis [50]. HFD-fed liver specific ASK1-knockout mice can experience higher degrees of hepatic damage and fibrosis, compared to controls. Moreover, liver-specific ASK1 over-expression protected mice from developing hepatic steatosis and fibrosis [51]. Whole-body ASK1-knockout mice are highly susceptible to lethal bacterial infection due to the blockage of autophagy in the liver. By suppressing ASK1 phosphorylation, dual-specificity phosphatase 9 (Dusp9) can prevent hepatic steatosis and fibrosis in mice with NASH [52]. These results exhibit that ASK1 is a potential therapeutic target for the management of liver fibrosis.

#### 3.2.2. Sirtuin 1 (SIRT1)

Sirtuin 1 (SIRT1), an NAD+-dependent enzyme involved in gene silencing by deacetylating histone and non-histone targets, has emerged as an essential metabolic sensor that coordinates changes in energy metabolism [53,54]. In liver tissue, SIRT1 promotes gluconeogenesis transcription through the activation of peroxisome proliferator-activated receptor γ coactivator 1-α (PGC1α) and forkhead box protein O1 (FOXO1) [55]. The deacetylation of liver X receptors (LXR) and farnesoid X receptor (FXR) by SIRT1 modulates cholesterol and bile acid homeostasis. SIRT1 also inhibits the expression of PPARγ and limits triglyceride synthesis in adipose tissue. Moreover, SIRT1 activators repressed the expression of SREBP target genes (*p* < 0.001), which play a crucial role in activating transcription of the key enzymes of fatty acid synthesis, such as FASN and ACC [56].

Clinical experiments showed that levels of SIRT1 were considerably decreased in the livers of NAFLD patients, while expression of the lipogenic genes was increased. Recent evidence has implicated that liver-specific SIRT1 knockout mice are susceptible to developing endoplasmic reticulum stress, hepatic inflammation, and hepatic steatosis. In contrast, overexpression of SIRT1 provided protection against high fat-induced hepatic steatosis in mice through the upregulation of gene expression enhancing fatty acid oxidation and downregulation of lipogenic gene expression. Resveratrol, an agonist of sirtuin 1, can significantly prevent HFD-induced insulin resistance and hepatic steatosis in rats via enhancing the activation of SIRT1 [57,58]. Several studies have reported that folic acid possessing the function of anti-steatosis and insulin sensing, may be related to the over-expression of gene SIRT1 [59,60,61].

#### 3.2.3. NLPR3

The activation of the NLPR3 inflammasome has been considered as a vital factor in the progress of NAFLD, especially as a modulator of progression from initial hepatic steatosis to NASH [62]. The NLRP3 inflammasome is an intracellular multiprotein complex associated with stimulation, producing mature IL-1β and inducing liver inflammation [63]. Two novel selective inhibitors of NLRP3, consisting of CY-09 and MCC950, can inhibit the level of caspase-1 and IL-1β and decrease the number of macrophages, as well as neutrophils in the liver, thus improving NAFLD pathology and fibrosis in obese diabetic mice [64]. Naringenin, a flavonoid compound possessing strong anti-inflammatory activity, was demonstrated to inhibit the development of NAFLD by downregulating the NLRP3 in hepatocytes, thus ameliorating inflammation in the livers of mice [65]. Targeting NLRP3 is a logical direction in the pharmacotherapy of NASH.

### 3.3. Potential Targets of Hepatic Fibrosis in NASH

#### 3.3.1. Lysyl Oxidase-like 2 (LOXL2)

Lysyl oxidase like-2 (LOXL2), a member of lysyl oxidase (LOX) family, possesses a conserved C-terminal amine oxidase domain, which consist of a His-X-His-X-His copper-binding motif and a lysine tyrosylquinone (LTQ) cofactor [66]. LOXL2 is able to catalyze the oxidative deamination of a ε-amino group of lysines and hydroxylysines in collagen and elastin, thereby promoting their polymerizations, which is essential for the tensile strength of the extracellular matrix (ECM) [67]. Loxl2 was predominantly expressed by hepatic stellate cells (HSCs), but it was also induced to a lesser extent in insulin resistant hepatocytes (HEPs) in response to lipotoxic damage [68].

In MCD-induced NASH mice, Loxl2 expression was strongly correlated with both the amount of steatosis and fibrosis severity [69]. A recent study suggested that hepatic *LoxL2* upregulation was specifically detected in NAFLD patients. Meanwhile LOXL2 expression can be induced by IR/T2D, and is associated with fibrosis severity in patients with NAFLD. In experimental models of liver damage, Loxl2 inhibition by monoclonal antibodies prevented liver fibrosis deposition and facilitated its reversal. PXS-5153A, a novel LOXL2 inhibitor, was capable of reducing LOXL2-mediated collagen oxidation and collagen crosslinking in vitro [70]. In HFD-induced liver fibrosis mouse models, PXS-5153A was able to diminish the severity of NAFLD and ameliorate liver function by reducing collagen content and collagen crosslinks (*p* < 0.001). These findings indicate that suppression of the enzymatic activities of LOXL2 may be an essential therapeutic strategy for the treatment of liver fibrosis.

#### 3.3.2. Vascular Adhesion Protein-1 (VAP-1)

VAP-1, an amine oxidase, has been shown to be beneficial in the management of hepatitis and liver fibrosis [71,72]. The serum soluble VAP-1 (sVAP-1) levels are raised in NAFLD patients compared with individuals in a control group [71]. The sVAP-1 with monoamine oxidase activity accounts for catalyzing the deamination of amines to yield the corresponding aldehyde and hydrogen peroxide. Hydrogen peroxide is a known adipocyte lipolysis inhibitor, and insulin mimic which increases hepatic glucose uptake as a fuel for de novo lipogenesis. Recent evidence suggests that the increase in liver inflammation, steatosis, and fibrosis in NAFLD patients is related to the ability of VAP-1 to support leukocyte recruitment, to prime hepatic glucose uptake, and to activate hepatic stellate cells [73]. A recent study exhibits that VAP-1 deficient mice show a reduction in intrahepatic CD4^+^ and NKT cell populations in response to methionine-choline deficient (MCD) diet-induced steatohepatitis (*p* < 0.001). Meanwhile, mice prophylactically treated with anti-VAP-1 antibody had significantly attenuated HFD-induced hepatic fibrosis. Furthermore, it is also reported that the increased expression of VAP-1 in hepatic fibrosis can promote leukocyte recruitment to the liver, which contributes to liver inflammation and fibrosis [74]. These findings suggest that VAP-1 is associated with inflammation and fibrosis in patients with NAFLD, indicating that VAP-1 may represent a valuable adjunct to other therapeutic approaches in NAFLD.

## 4. Potential Targeted Drugs for NAFLD

Currently, a variety of potential therapeutic strategies are used to evaluate the efficacy of different medications to treat NAFLD. Several drugs for treating other diseases, such as hypoglycemic drugs and hypolipidemic drugs, to an extent, are demonstrated to alleviate NAFLD. Clinical trials are currently used to check the pharmacological effect of NAFLD due to the lack of targeted drugs (Table 1).

### 4.1. Hypoglycemic Drugs

Glucagon-like peptide-1 (GLP-1) is an incretin hormone originated from the proglucagon polyprotein [75]. GLP-1 can increase the production of insulin secretion and decrease the secretion of glucagon from the pancreas, thus exhibiting a glucose-lowering action [76]. The secretion of GLP-1 is reduced in patients with NAFLD, indicating that GLP-1 can serve as a potential role in NAFLD treatment [77]. Liraglutide, a GLP-1 agonist, is capable of increasing insulin sensitivity and enhancing hepatic glucose uptake and glycogen synthesis [78]. Semaglutide, another GLP-1 agonist, has shown favorable effects on liver enzymes, insulin resistance, and hepatic fat content [79]. Tirzepatide, a synthetic GLP1/GIP peptide agonist, effectively reduced ALT and AST, increased adiponectin levels, resulting in significant improvement of liver fat content in T2DM sufferers with NASH [77]. Furthermore, the other GLP-1 agonists, including exenatide, lixisenatide, liraglutide, and dulaglutide, have exhibited significant antisteatotic activity.

### 4.2. Lipid-Lowering Drugs

Statins are HMG-CoA reductase inhibitors that serve as effective lipid-lowering drugs [80]. Statins can effectively reduce total plasma cholesterol and low-density lipoprotein levels [81]. They are often used to treat cardiovascular diseases, and conservatively treat NAFLD patients due to their hepatotoxicity. In NASH patients, statins significantly improved plasma transaminase levels, without causing liver injury [82].

### 4.3. THR-β Agonist

Thyroid hormone receptor-β (THR-β), primarily secreted from the liver, could improve metabolic disorders by enhancing fatty acid decomposition, thus reducing liver fat content [83]. MGL-3196 is an oral small-molecule THR-β agonist that can enhance liver cholesterol absorption and metabolism. When NASH patients were treated with MGL-3196 for 36 weeks, the levels of liver fat were significantly decreased [84]. VK2809, a THR-β agonist with lipid-lowering effects, is currently in phase 2–3 clinical trials for NASH. VK2809 can reduce LDL cholesterol and liver triglyceride levels by enhancing autophagy, mitochondrial biogenesis, and fatty acid β-oxidation [85]. At the same time, the safety and effectiveness of VK2809 for NASH treatment are being clinically evaluated [86]. Resmetirom, a liver THR-β receptor agonist, was demonstrated to ameliorate liver histology and decrease serum ALT and lipids in phase 2 clinical trial [84].

### 4.4. Vitamin E

Vitamin E is a prototypic antioxidant with the most profound anti-steatohepatitis effect when combined with other anti-NAFLD agents. It is capable of regulating the production of ROS and RNS and suppressing inflammation by downregulating the activation of transcription factor NF-κB [87,88]. The current guidelines by the American Association for the Study of Liver Disease (AASLD) have recommended the use of vitamin E in patients with biopsy-proven NASH and without diabetes. Currently, vitamin E, combined with pioglitazone, is a recommended therapy for NASH, and it usually improves liver histology significantly in NASH patients with T2D compared to placebo, although vitamin E alone had no similar effect [89]. Vitamin C, similar to vitamin E, with strong antioxidant properties, has been used to treat NASH and NAFLD in combination with vitamin E in several studies [90,91,92]. Despite the beneficial effects of vitamin E, vitamin E treatment can be associated with increased risk of all-cause mortality, prostate cancer, and hemorrhagic stroke [93,94]. 

### 4.5. FXR Agonist

The farnesoid X receptor (FXR), also known as the bile acid receptor, is a member of the nuclear receptor family mainly expressed in intestinal and liver tissues. FXR has emerged as an essential regulator in bile acid and glucose metabolism [95]. FXR activation has been shown to diminish hepatic lipogenesis, glucogenesis, cholesterol synthesis, and steatosis in animal models with NAFLD [96]. The whole-body FXR-knockout mice fed with a HFD show severe hepatic steatosis, necrotic inflammation, and fibrosis [97]. FXR agonists were demonstrated to inhibit the development of NASH in rodent models of HFD-induced NASH, where they also promoted the resolution of steatohepatitis and fibrosis [98]. Obeticholic acid (OCA), an FXR agonist, has been shown to decrease liver steatosis and fibrosis in preclinical models [99]. In phase 2 studies, OCA enhanced insulin sensitivity and reduced markers of liver damage in patients with T2DM and NAFLD [100]. Cilofexor (GS-9674) is a non-bile acid FXR agonist and is evaluated for the safety and tolerability of patients with NASH [101]. Other FXR agonists, including nidufexor, tropifexor, and EDP-305 have been developed and are currently in phase 2 trials [102]. 

### 4.6. PPAR Agonist

The peroxisome proliferator-activated receptors (PPARs) are members of the nuclear hormone receptor superfamily that regulate lipid metabolism by improving metabolic disorders [103]. Three PPARs, including α, δ, and γ, share the same target DNA sequence, but differ in ligand selectivity and tissue distribution [104]. Upon ligand binding, hepatic PPARα can induce mitochondrial FA uptake, β-oxidation, and ATP production. Several studies have shown that decreased hepatic PPARα expression was associated with insulin resistance and NASH severity [105]. Pemafibrate (K-877), a selective PPARα modulator, was demonstrated to improve liver pathology in the NASH mice model, and decrease serum transaminase activities and lipid profiles in patients with dyslipidemia [106].

PPARδ is capable of activating multiple metabolic pathways to regulate fatty acid β oxidation [107]. A mice model of NASH treated with PPARδ ligands achieves an upregulation of muscle oxidative capacity similar to that of exercise [108]. A novel PPARδ agonist, L-165041, has been shown to improve metabolic disorders by regulating inflammation and cholesterol efflux [107]. Seladelpar (MBX-8025), a selective PPARδ agonist, was evaluated in a phase 2a trial, and preliminary results showed strongly reduced hepatobiliary enzymes, lipid profiles, and high sensitivity C-reactive protein compared with a placebo [109]. Elafibranor, a dual PPARα/δ agonist treatment for NASH patients, was demonstrated to improve insulin sensitivity, metabolic disorders, and inflammation [110].

PPARγ also plays an essential role in human fat metabolism via regulating fat production and suppressing nuclear factor-kappa B (NF-κB) gene expression [111,112,113]. Recent experiments strongly suggest that PPARγ-deficient mice are at less risk of HFD-induced steatosis compared to normal mice [114]. Upregulated PPAR-γ expression was demonstrated to enhance lipid-induced macrophage polarization from the M1 phenotype towards the M2 phenotype, thus alleviating inflammation [115]. Saroglitazar, a PPARα/γ double agonist, has been shown to improve alanine aminotransferase levels and fatty liver in NAFLD patients with diabetic dyslipidemia, and it is approved to treat diabetic dyslipidemia in India [116]. Currently, saroglitazar has exhibited positive results and is in phase 3 clinical trials [117]. Lanifibranor, a PPARα/δ/γ agonist, can effectively inhibit liver inflammation and fibrosis, and it is currently being studied as a clinical trial drug for the treatment of liver fibrosis [118].

### 4.7. FGF19 Analogue

Fibroblast growth factor 19 (FGF19), a peptide hormone released after the activation of intestinal FXR, acts on the liver and leads to decreased gluconeogenesis in an insulin-independent manner. As a major downstream messenger in FXR and PPARα signaling, FGF19 is capable of inhibiting hepatic gluconeogenesis and promoting glycogen deposition [119]. FGF19 analogue aldafermin (NGM-282) is a recombinant variant of FGF-19 that reportedly retains its beneficial metabolic effect, but not the tumorigenic effects [120]. In a mouse model with NASH, NGM-282 treatment for three weeks noticeably decreased ALT and liver fat content, and resolved NASH [121].

### 4.8. FGF21 Analogue

Fibroblast growth factor 21 (FGF21) is a member of the hormone-like peptides secreted predominantly from the liver [122]. FGF-21 has emerged as a metabolic regulator, with insulin-sensitizing and antifibrotic properties [123,124]. It was also reported that FGF21-transfected mice induced by HFD reduced their body weight and their insulin resistance compared to those of normal mice [125]. Pegbelfermin (BMS-986036), a pegylated FGF-21 analogue with an increased half-life, was capable of improving insulin sensitivity, hepatic fat content, and de novo lipogenesis in mice models with NAFLD [126]. A phase 2 RDBPCT is assessing the effectiveness of BMS-986036 treatment on hepatic fat content in patients with NASH [127]. AKR-001 is a fusion protein that contains human IgG1-Fc with FGF21, and it is currently being assessed in a phase 2a trial after showing promising early safety and efficacy results [128].

### 4.9. CCR2/CCR5 Inhibitor

The C-C chemokine receptors 2/5 (CCR2/5) induce inflammation and aggravate NASH by promoting the recruitment of inflammatory cells to the liver [129]. Studies have shown that CCR2/5 promotes hepatic stellate cell activation after liver injury by increasing monocyte/macrophage recruitment and tissue infiltration, thus associating inflammation with liver fibrosis [130]. Zacarías et al. found that triazolopyrimidinone derivatives, a non- competitive intracellular antagonist of CC chemokine receptors 2 and 5, effectively inhibited β-arrestin recruitment in CCR5 and suppressed the inflammatory reaction [131]. Galectin-3 inhibitors can also suppress inflammatory macrophage recruitment by blocking chemokine receptor CCR2/5 and alleviating the subsequent fibrosis [132].

### 4.10. Pan-Caspase Inhibitor

Pan-caspase is a vital protease that regulates apoptosis, and is also involved in inflammatory reactions. Barreyro et al. found that a pan-caspase inhibitor, called emricasan (IDN-6556), can inhibit hepatocyte apoptosis in a NASH mouse model, thus reducing liver fibrosis and liver injury [133]. Shiffman et al. reported that a homocysteine protease inhibitor could suppress hepatocyte apoptosis in NAFLD patients [134]. Bral et al. found that pan-caspase inhibitors have inhibitory effects on hepatocyte apoptosis by reperfusion after hepatic ischemia in mice [135].

### 4.11. Galectin-3 Inhibitor

Galectin-3 is a unique chimeric β-galactoside binding protein of the galectin family, which is involved in fibrosis development in NASH patients [136]. In galectin-3 deficient mice with a high-fat diet, it was found that liver steatosis, liver injury, and liver fibrosis were significantly improved compared to wild-type mice [137]. GR-MD-02, a galectin-3 inhibitor, is one of the few drugs developed from the laboratory to clinical trials. GR-MD-02 has been shown to alleviate liver fibrosis associated with persistent liver injuries in the treatment of NASH patients with advanced fibrosis and is expected to be used as an anti-fibrosis drug to treat NAFLD [138,139].

**Table 1:** Mechanisms and primary outcomes of potential drugs for treating NAFLD.

Drug	Mechanism	Therapeutic Benefits	Side Effects	Clinical No.	Ref.
Metformin	Activating AMPK and inhibiting ACC	Inhibiting adipogenesis and improving IR	Appetite suppression	NCT00736385	[140]
Liraglutide	Activating GLP-1	Improving insulin sensitivity and metabolic disorders	Appetite suppression	NCT01237119	[141]
Statins	Inhibiting HMG-CoA	Reducing plasma total cholesterol and low density lipoprotein	Raised aminotransferases	NCT03434613	[142]
GS-0976	Inhibiting ACC	Reducing triglyceride accumulation in hepatocytes	Nausea and vomiting	NCT03987074	[143]
MGL-3196	Activating THR-β	Improving lipid metabolism and steatosis	Transient diarrhea	NCT04197479	[144]
Vitamin E	Inhibiting ROS	Reducing oxidative stress and inflammation			
Obeticholic acid	Activating FXR	Improving lipid metabolism	Pruritus	NCT01265498	[145]
Cilofexor	Activating FXR	Improving inflammation and fibrosis	Pruritus	NCT02654002	[146]
Tropifexor	Activating FXR	Improving adipogenesis, inflammation, and fibrosis	Pruritus and cholestatic disorders	NCT03681457	[147]
Elafibranor	Activating PPARα/δ	Improving inflammation and fibrosis	Pruritus	NCT01694849	[148]
Lanifibranor	Activating PPARα/δ/γ	Improving NASH and liver fibrosis	peripheral edema	NCT03459079	[149]
NGM282	Activating FGF19	Reducing liver fat, liver injury, and inflammation	Nausea and abdominal pain	NCT02443116	[150]
BMS-986036	Activating FGF21	Improving insulin sensitivity, liver fat content, and adiponectin content	Immunogenicity	NCT03486899	[151]
Cenicriviroc	Inhibiting CCR2/CCR5	Improving inflammation and fibrosis	Headache	NCT02330549	[152]
IDN-6556	Inhibiting pan-caspase	Improving apoptosis, inflammation, and fibrosis		NCT02077374	[153]
GR-MD-02	Inhibiting galectin-3	Improving fibrosis		NCT02077374	[154]
Empagliflozin	Inhibiting SGLT-2	Reducing ALT and liver fat	Acute kidney injury		[155]
Canagliflozin	Inhibiting SGLT-2	Improving AST, FIB-4 index	Acute renal failure		[156]
Rosiglitazone	Activating PPAR-γ	Improving steatosis and transaminase levels	Heart failure and peripheral edema		[157]
Pioglitazone	Activating PPAR-γ	Improving steatosis, inflammation, and liver histology	Hypoglycemia and lower limb edema	NCT00063622	[158]
Semaglutide	Activating GLP-1	Reducing body weight and liver enzymes	Nausea and diarrhea	NCT02453711	[159]
Pentoxifylline	Inhibiting TNF-a	Improving liver enzymes and insulin resistance	Nausea and vomiting		
JKB-121	Activating TLR-4	Reducing liver fat content	Mild drug-related adverse events	NCT02442687	[160]
Emricasan	Inhibiting caspase	Improving fibrosis	Chest pain and headache	NCT02686762	[134]
Selonsertib	Inhibiting ASK-1	Improving fibrosis and reduction in hepatic decompensation, hepatocellular carcinoma	Mild drug-related adverse events	NCT03053063	[161]
Atorvastatin	Inhibiting HMG-CoA	Reducing steatosis and improving liver density	Autoimmune hepatitis		[162]
Ezetimibe	Decreasing intestinal cholesterol absorption	Improving aminotransferases and hepatocyte ballooning	New-onset diabetes and increased HbA1c levels		[163]
GS-9674	Activating FXR	Reducing hepatic fat and improving liver biochemistry		NCT02854605	[164]
Aramchol	Inhibiting SCD-1	Reducing liver fat, Ballooning, and AST		NCT04104321	[165]
Losartan	Activating TGF-β	Improving serum aminotransferases and histologic outcomes	Angioedema		[166]
Telmisartan	Inhibiting CCR2 and CCR5	Reducing serum ALT levels and improving insulin sensitivity steatosis	Angioedema	NCT01088295	[167,168]
VK-2809	Activating thyroid receptor β	Reducing fat in liver			[144]
Simtuzumab	Monoclonal antibody of LOXL2	Improving liver cirrhosis		NCT01672866	[152]

## 5. Discussion

There are currently no FDA approved medications for the treatment of NAFLD/NASH. Current management for NAFLD includes diet and lifestyle modifications for achieving weight loss, management of underlying metabolic risk factors, and pharmacological strategies, where there is evidence of NASH or of advanced fibrosis [169]. However, it is difficult to achieve long-term compliance to dietary restriction, unless cardiovascular comorbidities are present. Several medications, such as pioglitazone, obeticholic acid, and vitamin E, remain the strategy for disease management in patients with NAFLD [170]. However, it is only effective in part of the patients, suggesting that additional treatments are still urgently needed, and that subgroups may be present in NAFLD, responding differently to various treatment strategies.

Thus, a combination of medications was required for addressing different aspects of particular NAFLD phenotype. In this paper, the latest upcoming pharmacological targets for this disease were reviewed, highlighting in detail the most novel treatment strategies, as well as currently marketed drugs. Meanwhile, the progress on the pathogenesis of NAFLD was well described, such as gut–liver axis, which may lead to many novel therapies targeting gut dysbiosis in the future.

## 6. Conclusions

Recently, significant progress has been made in understanding the pathogenesis of NAFLD and the subsequent development of drugs against the disease [171]. NAFLD may be caused by genetic susceptibility, diet, intestinal microbes, and other factors. It begins with excessive fat production in the liver that results in the accumulation of a large amount of fat, leading to liver cell degeneration, inflammation, and a fibrotic process involving interactions among many proteins [172]. Several drugs have been shown to alleviate NAFLD by acting on these proteins. Some drugs target various aspects of NAFLD, such as lipogenesis and steatosis. Therefore, additional in-depth studies are needed to develop comprehensive and effective drugs, which can comprehensively prevent lipotoxicity, steatosis, oxidative stress, inflammation, and hepatocyte apoptosis.

## Data Availability

Not applicable.

## References

[1] Marchisello S., Di Pino A., Scicali R., Urbano F., Piro S., Purrello F., Rabuazzo A.M. (2019). Pathophysiological, Molecular and Therapeutic Issues of Nonalcoholic Fatty Liver Disease: An Overview. Int. J. Mol. Sci..

[2] Angulo P. (2002). Nonalcoholic fatty liver disease. N. Engl. J. Med..

[3] Younossi Z., Anstee Q.M., Marietti M., Hardy T., Henry L., Eslam M., George J., Bugianesi E. (2018). Global burden of NAFLD and NASH: Trends, predictions, risk factors and prevention. Nat. Rev. Gastroenterol. Hepatol..

[4] Alvarez C.S., Graubard B.I., Thistle J.E., Petrick J.L., McGlynn K.A. (2019). Attributable Fractions of NAFLD for Mortality in the United States: Results From NHANES III With 27 Years of Follow-up. Hepatology.

[5] Younossi Z., Tacke F., Arrese M., Chander Sharma B., Mostafa I., Bugianesi E., Wai-Sun Wong V., Yilmaz Y., George J., Fan J. (2019). Global Perspectives on Nonalcoholic Fatty Liver Disease and Nonalcoholic Steatohepatitis. Hepatology.

[6] Li J., Zou B., Yeo Y.H., Feng Y., Xie X., Lee D.H., Fujii H., Wu Y., Kam L.Y., Ji F. (2019). Prevalence, incidence, and outcome of non-alcoholic fatty liver disease in Asia, 1999-2019: A systematic review and meta-analysis. Lancet Gastroenterol. Hepatol..

[7] Rehm J., Mathers C., Popova S., Thavorncharoensap M., Teerawattananon Y., Patra J. (2009). Global burden of disease and injury and economic cost attributable to alcohol use and alcohol-use disorders. Lancet.

[8] Calzadilla Bertot L., Adams L.A. (2016). The Natural Course of Non-Alcoholic Fatty Liver Disease. Int. J. Mol. Sci..

[9] Eslam M., Valenti L., Romeo S. (2018). Genetics and epigenetics of NAFLD and NASH: Clinical impact. J. Hepatol..

[10] Cohen J.C., Horton J.D., Hobbs H.H. (2011). Human fatty liver disease: Old questions and new insights. Science.

[11] Targher G., Byrne C.D., Lonardo A., Zoppini G., Barbui C. (2016). Non-alcoholic fatty liver disease and risk of incident cardiovascular disease: A meta-analysis. J. Hepatol..

[12] Lonardo A., Nascimbeni F., Mantovani A., Targher G. (2018). Hypertension, diabetes, atherosclerosis and NASH: Cause or consequence?. J. Hepatol..

[13] Day C.P., James O.F. (1998). Steatohepatitis: A tale of two “hits”?. Gastroenterology.

[14] Friedman S.L., Neuschwander-Tetri B.A., Rinella M., Sanyal A.J. (2018). Mechanisms of NAFLD development and therapeutic strategies. Nat. Med..

[15] Buzzetti E., Pinzani M., Tsochatzis E.A. (2016). The multiple-hit pathogenesis of non-alcoholic fatty liver disease (NAFLD). Metabolism.

[16] Marra F., Svegliati-Baroni G. (2018). Lipotoxicity and the gut-liver axis in NASH pathogenesis. J. Hepatol..

[17] Byrne C.D., Targher G. (2020). What’s new in NAFLD pathogenesis, biomarkers and treatment?. Nat. Rev. Gastroenterol. Hepatol..

[18] Tsuchida T., Friedman S.L. (2017). Mechanisms of hepatic stellate cell activation. Nat. Rev. Gastroenterol. Hepatol..

[19] Tacke F., Weiskirchen R. (2021). Non-alcoholic fatty liver disease (NAFLD)/non-alcoholic steatohepatitis (NASH)-related liver fibrosis: Mechanisms, treatment and prevention. Ann. Transl. Med..

[20] Zhou W.C., Zhang Q.B., Qiao L. (2014). Pathogenesis of liver cirrhosis. World J. Gastroenterol..

[21] Tilg H., Adolph T.E., Moschen A.R. (2021). Multiple Parallel Hits Hypothesis in Nonalcoholic Fatty Liver Disease: Revisited After a Decade. Hepatology.

[22] Makri E., Goulas A., Polyzos S.A. (2021). Epidemiology, Pathogenesis, Diagnosis and Emerging Treatment of Nonalcoholic Fatty Liver Disease. Arch. Med. Res..

[23] Fujii H., Kawada N., Japan Study Group Of Nafld J.-N. (2020). The Role of Insulin Resistance and Diabetes in Nonalcoholic Fatty Liver Disease. Int. J. Mol. Sci..

[24] Koh A., Molinaro A., Ståhlman M., Khan M.T., Schmidt C., Mannerås-Holm L., Wu H., Carreras A., Jeong H., Olofsson L.E. (2018). Microbially Produced Imidazole Propionate Impairs Insulin Signaling through mTORC1. Cell.

[25] Zhao M., Zhao L., Xiong X., He Y., Huang W., Liu Z., Ji L., Pan B., Guo X., Wang L. (2020). TMAVA, a Metabolite of Intestinal Microbes, Is Increased in Plasma From Patients With Liver Steatosis, Inhibits γ-Butyrobetaine Hydroxylase, and Exacerbates Fatty Liver in Mice. Gastroenterology.

[26] Nati M., Chung K.J., Chavakis T. (2022). The Role of Innate Immune Cells in Nonalcoholic Fatty Liver Disease. J. Innate Immun..

[27] Ala M. (2021). SGLT2 Inhibition for Cardiovascular Diseases, Chronic Kidney Disease, and NAFLD. Endocrinology.

[28] Sumida Y., Yoneda M. (2018). Current and future pharmacological therapies for NAFLD/NASH. J. Gastroenterol..

[29] Chiang H., Lee J.C., Huang H.C., Huang H., Liu H.K., Huang C. (2020). Delayed intervention with a novel SGLT2 inhibitor NGI001 suppresses diet-induced metabolic dysfunction and non-alcoholic fatty liver disease in mice. Br. J. Pharm..

[30] Xu L., Nagata N., Nagashimada M., Zhuge F., Ni Y., Chen G., Mayoux E., Kaneko S., Ota T. (2017). SGLT2 Inhibition by Empagliflozin Promotes Fat Utilization and Browning and Attenuates Inflammation and Insulin Resistance by Polarizing M2 Macrophages in Diet-induced Obese Mice. EBioMedicine.

[31] Trauner M., Fuchs C.D. (2022). Novel therapeutic targets for cholestatic and fatty liver disease. Gut.

[32] Libby A.E., Bales E., Orlicky D.J., McManaman J.L. (2016). Perilipin-2 Deletion Impairs Hepatic Lipid Accumulation by Interfering with Sterol Regulatory Element-binding Protein (SREBP) Activation and Altering the Hepatic Lipidome. J. Biol. Chem..

[33] Najt C.P., Senthivinayagam S., Aljazi M.B., Fader K.A., Olenic S.D., Brock J.R., Lydic T.A., Jones A.D., Atshaves B.P. (2016). Liver-specific loss of Perilipin 2 alleviates diet-induced hepatic steatosis, inflammation, and fibrosis. Am. J. Physiol. Gastrointest. Liver Physiol..

[34] Mitro N., Mak P.A., Vargas L., Godio C., Hampton E., Molteni V., Kreusch A., Saez E. (2007). The nuclear receptor LXR is a glucose sensor. Nature.

[35] Zhou J., Febbraio M., Wada T., Zhai Y., Kuruba R., He J., Lee J.H., Khadem S., Ren S., Li S. (2008). Hepatic fatty acid transporter Cd36 is a common target of LXR, PXR, and PPARgamma in promoting steatosis. Gastroenterology.

[36] Huang P., Kaluba B., Jiang X.L., Chang S., Tang X.F., Mao L.F., Zhang Z.P., Huang F.Z. (2018). Liver X Receptor Inverse Agonist SR9243 Suppresses Nonalcoholic Steatohepatitis Intrahepatic Inflammation and Fibrosis. Biomed. Res. Int..

[37] Kim W., Kim B.G., Lee J.S., Lee C.K., Yeon J.E., Chang M.S., Kim J.H., Kim H., Yi S., Lee J. (2017). Randomised clinical trial: The efficacy and safety of oltipraz, a liver X receptor alpha-inhibitory dithiolethione in patients with non-alcoholic fatty liver disease. Aliment. Pharm. Ther..

[38] Harriman G., Greenwood J., Bhat S., Huang X., Wang R., Paul D., Tong L., Saha A.K., Westlin W.F., Kapeller R. (2016). Acetyl-CoA carboxylase inhibition by ND-630 reduces hepatic steatosis, improves insulin sensitivity, and modulates dyslipidemia in rats. Proc. Natl. Acad. Sci. USA.

[39] Alkhouri N., Lawitz E., Noureddin M., DeFronzo R., Shulman G.I. (2020). GS-0976 (Firsocostat): An investigational liver-directed acetyl-CoA carboxylase (ACC) inhibitor for the treatment of non-alcoholic steatohepatitis (NASH). Expert Opin. Investig. Drugs.

[40] Lawitz E.J., Coste A., Poordad F., Alkhouri N., Loo N., McColgan B.J., Tarrant J.M., Nguyen T., Han L., Chung C. (2018). Acetyl-CoA Carboxylase Inhibitor GS-0976 for 12 Weeks Reduces Hepatic De Novo Lipogenesis and Steatosis in Patients With Nonalcoholic Steatohepatitis. Clin. Gastroenterol. Hepatol..

[41] Beysen C., Schroeder P., Wu E., Brevard J., Ribadeneira M., Lu W., Dole K., O’Reilly T., Morrow L., Hompesch M. (2021). Inhibition of fatty acid synthase with FT-4101 safely reduces hepatic de novo lipogenesis and steatosis in obese subjects with non-alcoholic fatty liver disease: Results from two early-phase randomized trials. Diabetes Obes. Metab..

[42] Loomba R., Mohseni R., Lucas K.J., Gutierrez J.A., Perry R.G., Trotter J.F., Rahimi R.S., Harrison S.A., Ajmera V., Wayne J.D. (2021). TVB-2640 (FASN Inhibitor) for the Treatment of Nonalcoholic Steatohepatitis: FASCINATE-1, a Randomized, Placebo-Controlled Phase 2a Trial. Gastroenterology.

[43] Gaballah A.H., Bingham K., Hammoud G.M., Kemble G., Buckley D., McCulloch W., Manrique-Acevedo C. (2020). Fatty Acid Synthase Inhibitor TVB-2640 Reduces Hepatic de Novo Lipogenesis in Males With Metabolic Abnormalities. Hepatology.

[44] Zhang M., Tang Y., Tang E., Lu W. (2020). MicroRNA-103 represses hepatic de novo lipogenesis and alleviates NAFLD via targeting FASN and SCD1. Biochem. Biophys. Res. Commun..

[45] Day E.A., Ford R.J., Steinberg G.R. (2017). AMPK as a Therapeutic Target for Treating Metabolic Diseases. Trends Endocrinol. Metab..

[46] Boudaba N., Marion A., Huet C., Pierre R., Viollet B., Foretz M. (2018). AMPK Re-Activation Suppresses Hepatic Steatosis but its Downregulation Does Not Promote Fatty Liver Development. EBioMedicine.

[47] Zhu X., Bian H., Wang L., Sun X., Xu X., Yan H., Xia M., Chang X., Lu Y., Li Y. (2019). Berberine attenuates nonalcoholic hepatic steatosis through the AMPK-SREBP-1c-SCD1 pathway. Free Radic. Biol. Med..

[48] Shang J., Chen L.L., Xiao F.X., Sun H., Ding H.C., Xiao H. (2008). Resveratrol improves non-alcoholic fatty liver disease by activating AMP-activated protein kinase. Acta Pharm. Sin..

[49] Qiang X., Xu L., Zhang M., Zhang P., Wang Y., Wang Y., Zhao Z., Chen H., Liu X., Zhang Y. (2016). Demethyleneberberine attenuates non-alcoholic fatty liver disease with activation of AMPK and inhibition of oxidative stress. Biochem. Biophys. Res. Commun..

[50] Sakauchi C., Wakatsuki H., Ichijo H., Hattori K. (2017). Pleiotropic properties of ASK1. Biochim. Biophys. Acta Gen. Subj..

[51] Challa T.D., Wueest S., Lucchini F.C., Dedual M., Modica S., Borsigova M., Wolfrum C., Blüher M., Konrad D. (2019). Liver ASK1 protects from non-alcoholic fatty liver disease and fibrosis. EMBO Mol. Med..

[52] Ye P., Xiang M., Liao H., Liu J., Luo H., Wang Y., Huang L., Chen M., Xia J. (2019). Dual-Specificity Phosphatase 9 Protects Against Nonalcoholic Fatty Liver Disease in Mice Through ASK1 Suppression. Hepatology.

[53] Zhang J., Peng J., Kong D., Wang X., Wang Z., Liu J., Yu W., Wu H., Long Z., Zhang W. (2021). Silent information regulator 1 suppresses epithelial-to-mesenchymal transition in lung cancer cells via its regulation of mitochondria status. Life Sci..

[54] Silva J.P., Wahlestedt C. (2010). Role of Sirtuin 1 in metabolic regulation. Drug Discov. Today.

[55] You M., Jogasuria A., Taylor C., Wu J. (2015). Sirtuin 1 signaling and alcoholic fatty liver disease. Hepatobiliary Surg. Nutr..

[56] Wu T., Liu Y.H., Fu Y.C., Liu X.M., Zhou X.H. (2014). Direct evidence of sirtuin downregulation in the liver of non-alcoholic fatty liver disease patients. Ann. Clin. Lab. Sci..

[57] Haohao Z., Guijun Q., Juan Z., Wen K., Lulu C. (2015). Resveratrol improves high-fat diet induced insulin resistance by rebalancing subsarcolemmal mitochondrial oxidation and antioxidantion. J. Physiol. Biochem..

[58] Côté C.D., Rasmussen B.A., Duca F.A., Zadeh-Tahmasebi M., Baur J.A., Daljeet M., Breen D.M., Filippi B.M., Lam T.K. (2015). Resveratrol activates duodenal Sirt1 to reverse insulin resistance in rats through a neuronal network. Nat. Med..

[59] Xin F.Z., Zhao Z.H., Zhang R.N., Pan Q., Gong Z.Z., Sun C., Fan J.G. (2020). Folic acid attenuates high-fat diet-induced steatohepatitis via deacetylase SIRT1-dependent restoration of PPARα. World J. Gastroenterol..

[60] Leclerc D., Jelinek J., Christensen K.E., Issa J.J., Rozen R. (2021). High folic acid intake increases methylation-dependent expression of Lsr and dysregulates hepatic cholesterol homeostasis. J. Nutr. Biochem..

[61] Salman M., Kamel M.A., El-Nabi S.E.H., Ismail A.H.A., Ullah S., Al-Ghamdi A., Hathout H.M.R., El-Garawani I.M. (2022). The regulation of HBP1, SIRT1, and SREBP-1c genes and the related microRNAs in non-alcoholic fatty liver rats: The association with the folic acid anti-steatosis. PLoS ONE.

[62] Yu L., Hong W., Lu S., Li Y., Guan Y., Weng X., Feng Z. (2022). The NLRP3 Inflammasome in Non-Alcoholic Fatty Liver Disease and Steatohepatitis: Therapeutic Targets and Treatment. Front. Pharm..

[63] Mridha A.R., Wree A., Robertson A.A.B., Yeh M.M., Johnson C.D., Van Rooyen D.M., Haczeyni F., Teoh N.C., Savard C., Ioannou G.N. (2017). NLRP3 inflammasome blockade reduces liver inflammation and fibrosis in experimental NASH in mice. J. Hepatol..

[64] Qu J., Yuan Z., Wang G., Wang X., Li K. (2019). The selective NLRP3 inflammasome inhibitor MCC950 alleviates cholestatic liver injury and fibrosis in mice. Int. Immunopharmacol..

[65] Wang Q., Ou Y., Hu G., Wen C., Yue S., Chen C., Xu L., Xie J., Dai H., Xiao H. (2020). Naringenin attenuates non-alcoholic fatty liver disease by down-regulating the NLRP3/NF-κB pathway in mice. Br. J. Pharm..

[66] Barry-Hamilton V., Spangler R., Marshall D., McCauley S., Rodriguez H.M., Oyasu M., Mikels A., Vaysberg M., Ghermazien H., Wai C. (2010). Allosteric inhibition of lysyl oxidase-like-2 impedes the development of a pathologic microenvironment. Nat. Med..

[67] Puente A., Fortea J.I., Cabezas J., Arias Loste M.T., Iruzubieta P., Llerena S., Huelin P., Fábrega E., Crespo J. (2019). LOXL2-A New Target in Antifibrogenic Therapy?. Int. J. Mol. Sci..

[68] Dongiovanni P., Meroni M., Baselli G.A., Bassani G.A., Rametta R., Pietrelli A., Maggioni M., Facciotti F., Trunzo V., Badiali S. (2017). Insulin resistance promotes Lysyl Oxidase Like 2 induction and fibrosis accumulation in non-alcoholic fatty liver disease. Clin. Sci..

[69] Moon H.J., Finney J., Ronnebaum T., Mure M. (2014). Human lysyl oxidase-like 2. Bioorganic Chem..

[70] Schilter H., Findlay A.D., Perryman L., Yow T.T., Moses J., Zahoor A., Turner C.I., Deodhar M., Foot J.S., Zhou W. (2019). The lysyl oxidase like 2/3 enzymatic inhibitor, PXS-5153A, reduces crosslinks and ameliorates fibrosis. J. Cell Mol. Med..

[71] Weston C.J., Shepherd E.L., Claridge L.C., Rantakari P., Curbishley S.M., Tomlinson J.W., Hubscher S.G., Reynolds G.M., Aalto K., Anstee Q.M. (2015). Vascular adhesion protein-1 promotes liver inflammation and drives hepatic fibrosis. J. Clin. Investig..

[72] Öksüz Z., Üçbilek E., Serin M.S., Yaraş S., Temel G.O., Sezgin O. (2020). Circulating vascular adhesion protein-1(VAP-1): A possible biomarker for liver fibrosis associated with chronic hepatitis B and C. Braz. J. Microbiol. Publ. Braz. Soc. Microbiol..

[73] Shepherd E.L., Karim S., Newsome P.N., Lalor P.F. (2020). Inhibition of vascular adhesion protein-1 modifies hepatic steatosis in vitro and in vivo. World J. Hepatol..

[74] Trivedi P.J., Tickle J., Vesterhus M.N., Eddowes P.J., Bruns T., Vainio J., Parker R., Smith D., Liaskou E., Thorbjørnsen L.W. (2018). Vascular adhesion protein-1 is elevated in primary sclerosing cholangitis, is predictive of clinical outcome and facilitates recruitment of gut-tropic lymphocytes to liver in a substrate-dependent manner. Gut.

[75] Holst J.J., Jepsen S.L., Modvig I. (2022). GLP-1—Incretin and pleiotropic hormone with pharmacotherapy potential. Increasing secretion of endogenous GLP-1 for diabetes and obesity therapy. Curr. Opin. Pharmacol..

[76] Mathiesen D.S., Bagger J.I., Bergmann N.C., Lund A., Christensen M.B., Vilsbøll T., Knop F.K. (2019). The Effects of Dual GLP-1/GIP Receptor Agonism on Glucagon Secretion-A Review. Int. J. Mol. Sci..

[77] Hartman M.L., Sanyal A.J., Loomba R., Wilson J.M., Nikooienejad A., Bray R., Karanikas C.A., Duffin K.L., Robins D.A., Haupt A. (2020). Effects of Novel Dual GIP and GLP-1 Receptor Agonist Tirzepatide on Biomarkers of Nonalcoholic Steatohepatitis in Patients With Type 2 Diabetes. Diabetes Care.

[78] Qin Y., Chen M., Yang Y., Zhou X.R., Shao S.Y., Wang D.W., Yuan G. (2018). Liraglutide improves hepatic insulin resistance via the canonical Wnt signaling pathway. Mol. Med. Rep..

[79] McCrimmon R.J., Catarig A.M., Frias J.P., Lausvig N.L., le Roux C.W., Thielke D., Lingvay I. (2020). Effects of once-weekly semaglutide vs once-daily canagliflozin on body composition in type 2 diabetes: A substudy of the SUSTAIN 8 randomised controlled clinical trial. Diabetologia.

[80] Sirtori C.R. (2014). The pharmacology of statins. Pharm. Res..

[81] Nascimbeni F., Pellegrini E., Lugari S., Mondelli A., Bursi S., Onfiani G., Carubbi F., Lonardo A. (2019). Statins and nonalcoholic fatty liver disease in the era of precision medicine: More friends than foes. Atherosclerosis.

[82] Bril F., Portillo Sanchez P., Lomonaco R., Orsak B., Hecht J., Tio F., Cusi K. (2017). Liver Safety of Statins in Prediabetes or T2DM and Nonalcoholic Steatohepatitis: Post Hoc Analysis of a Randomized Trial. J. Clin. Endocrinol. Metab..

[83] Kannt A., Wohlfart P., Madsen A.N., Veidal S.S., Feigh M., Schmoll D. (2021). Activation of thyroid hormone receptor-β improved disease activity and metabolism independent of body weight in a mouse model of non-alcoholic steatohepatitis and fibrosis. Br. J. Pharm..

[84] Harrison S.A., Bashir M.R., Guy C.D., Zhou R., Moylan C.A., Frias J.P., Alkhouri N., Bansal M.B., Baum S., Neuschwander-Tetri B.A. (2019). Resmetirom (MGL-3196) for the treatment of non-alcoholic steatohepatitis: A multicentre, randomised, double-blind, placebo-controlled, phase 2 trial. Lancet.

[85] Zhou J., Waskowicz L.R., Lim A., Liao X.H., Lian B., Masamune H., Refetoff S., Tran B., Koeberl D.D., Yen P.M. (2019). A Liver-Specific Thyromimetic, VK2809, Decreases Hepatosteatosis in Glycogen Storage Disease Type Ia. Thyroid Off. J. Am. Thyroid Assoc..

[86] Luong X.G., Stevens S.K., Jekle A., Lin T.I., Gupta K., Misner D., Chanda S., Mukherjee S., Williams C., Stoycheva A. (2020). Regulation of gene transcription by thyroid hormone receptor β agonists in clinical development for the treatment of non-alcoholic steatohepatitis (NASH). PLoS ONE.

[87] Lee G.Y., Han S.N. (2018). The Role of Vitamin E in Immunity. Nutrients.

[88] Peh H.Y., Tan W.S., Liao W., Wong W.S. (2016). Vitamin E therapy beyond cancer: Tocopherol versus tocotrienol. Pharmacol. Ther..

[89] Bril F., Biernacki D.M., Kalavalapalli S., Lomonaco R., Subbarayan S.K., Lai J., Tio F., Suman A., Orsak B.K., Hecht J. (2019). Role of Vitamin E for Nonalcoholic Steatohepatitis in Patients With Type 2 Diabetes: A Randomized Controlled Trial. Diabetes Care.

[90] Kawanaka M., Nishino K., Nakamura J., Suehiro M., Goto D., Urata N., Oka T., Kawamoto H., Nakamura H., Yodoi J. (2013). Treatment of nonalcoholic steatohepatitis with vitamins E and C: A pilot study. Hepatic Med. Evid. Res..

[91] Murer S.B., Aeberli I., Braegger C.P., Gittermann M., Hersberger M., Leonard S.W., Taylor A.W., Traber M.G., Zimmermann M.B. (2014). Antioxidant supplements reduced oxidative stress and stabilized liver function tests but did not reduce inflammation in a randomized controlled trial in obese children and adolescents. J. Nutr..

[92] Perumpail B.J., Li A.A., John N., Sallam S., Shah N.D., Kwong W., Cholankeril G., Kim D., Ahmed A. (2018). The Role of Vitamin E in the Treatment of NAFLD. Diseases.

[93] Schürks M., Glynn R.J., Rist P.M., Tzourio C., Kurth T. (2010). Effects of vitamin E on stroke subtypes: Meta-analysis of randomised controlled trials. BMJ Clin. Res. Ed..

[94] Oliver C.J., Myers S.P. (2017). Validity of a Cochrane Systematic Review and meta-analysis for determining the safety of vitamin E. BMC Complementary Altern. Med..

[95] Massafra V., van Mil S.W.C. (2018). Farnesoid X receptor: A “homeostat” for hepatic nutrient metabolism. Biochim. Biophys. Acta Mol. Basis Dis..

[96] Sun L., Pang Y., Wang X., Wu Q., Liu H., Liu B., Liu G., Ye M., Kong W., Jiang C. (2019). Ablation of gut microbiota alleviates obesity-induced hepatic steatosis and glucose intolerance by modulating bile acid metabolism in hamsters. Acta Pharm. Sin. B.

[97] Pierre J.F., Li Y., Gomes C.K., Rao P., Chang E.B., Yin D.P. (2019). Bile Diversion Improves Metabolic Phenotype Dependent on Farnesoid X Receptor (FXR). Obesity.

[98] Hernandez E.D., Zheng L., Kim Y., Fang B., Liu B., Valdez R.A., Dietrich W.F., Rucker P.V., Chianelli D., Schmeits J. (2019). Tropifexor-Mediated Abrogation of Steatohepatitis and Fibrosis Is Associated With the Antioxidative Gene Expression Profile in Rodents. Hepatol. Commun..

[99] Tølbøl K.S., Kristiansen M.N., Hansen H.H., Veidal S.S., Rigbolt K.T., Gillum M.P., Jelsing J., Vrang N., Feigh M. (2018). Metabolic and hepatic effects of liraglutide, obeticholic acid and elafibranor in diet-induced obese mouse models of biopsy-confirmed nonalcoholic steatohepatitis. World J. Gastroenterol..

[100] Mudaliar S., Henry R.R., Sanyal A.J., Morrow L., Marschall H.U., Kipnes M., Adorini L., Sciacca C.I., Clopton P., Castelloe E. (2013). Efficacy and safety of the farnesoid X receptor agonist obeticholic acid in patients with type 2 diabetes and nonalcoholic fatty liver disease. Gastroenterology.

[101] Patel K., Harrison S.A., Elkhashab M., Trotter J.F., Herring R., Rojter S.E., Kayali Z., Wong V.W., Greenbloom S., Jayakumar S. (2020). Cilofexor, a Nonsteroidal FXR Agonist, in Patients With Noncirrhotic NASH: A Phase 2 Randomized Controlled Trial. Hepatology.

[102] Fiorucci S., Biagioli M., Sepe V., Zampella A., Distrutti E. (2020). Bile acid modulators for the treatment of nonalcoholic steatohepatitis (NASH). Expert Opin. Investig. Drugs.

[103] Broeders N., Abramowicz D. (2002). Peroxisome proliferator-activated receptors (PPARs): Novel therapeutic targets in renal disease. Kidney Int..

[104] Schoonjans K., Staels B., Auwerx J. (1996). The peroxisome proliferator activated receptors (PPARS) and their effects on lipid metabolism and adipocyte differentiation. Biochim. Biophys. Acta.

[105] Francque S., Verrijken A., Caron S., Prawitt J., Paumelle R., Derudas B., Lefebvre P., Taskinen M.R., Van Hul W., Mertens I. (2015). PPARα gene expression correlates with severity and histological treatment response in patients with non-alcoholic steatohepatitis. J. Hepatol..

[106] Sairyo M., Kobayashi T., Masuda D., Kanno K., Zhu Y., Okada T., Koseki M., Ohama T., Nishida M., Sakata Y. (2018). A Novel Selective PPARα Modulator (SPPARMα), K-877 (Pemafibrate), Attenuates Postprandial Hypertriglyceridemia in Mice. J. Atheroscler. Thromb..

[107] Lim H.J., Park J.H., Lee S., Choi H.E., Lee K.S., Park H.Y. (2009). PPARdelta ligand L-165041 ameliorates Western diet-induced hepatic lipid accumulation and inflammation in LDLR-/- mice. Eur. J. Pharm..

[108] Gan Z., Burkart-Hartman E.M., Han D.H., Finck B., Leone T.C., Smith E.Y., Ayala J.E., Holloszy J., Kelly D.P. (2011). The nuclear receptor PPARβ/δ programs muscle glucose metabolism in cooperation with AMPK and MEF2. Genes Dev..

[109] Haczeyni F., Wang H., Barn V., Mridha A.R., Yeh M.M., Haigh W.G., Ioannou G.N., Choi Y.J., McWherter C.A., Teoh N.C. (2017). The selective peroxisome proliferator-activated receptor-delta agonist seladelpar reverses nonalcoholic steatohepatitis pathology by abrogating lipotoxicity in diabetic obese mice. Hepatol. Commun.

[110] Westerouen Van Meeteren M.J., Drenth J.P.H., Tjwa E. (2020). Elafibranor: A potential drug for the treatment of nonalcoholic steatohepatitis (NASH). Expert Opin. Investig. Drugs.

[111] Pascual G., Fong A.L., Ogawa S., Gamliel A., Li A.C., Perissi V., Rose D.W., Willson T.M., Rosenfeld M.G., Glass C.K. (2005). A SUMOylation-dependent pathway mediates transrepression of inflammatory response genes by PPAR-gamma. Nature.

[112] Ricote M., Huang J.T., Welch J.S., Glass C.K. (1999). The peroxisome proliferator-activated receptor(PPARgamma) as a regulator of monocyte/macrophage function. J. Leukoc. Biol..

[113] Konstantinopoulos P.A., Vandoros G.P., Sotiropoulou-Bonikou G., Kominea A., Papavassiliou A.G. (2007). NF-kappaB/PPAR gamma and/or AP-1/PPAR gamma ‘on/off’ switches and induction of CBP in colon adenocarcinomas: Correlation with COX-2 expression. Int. J. Colorectal Dis..

[114] Wu C.W., Chu E.S., Lam C.N., Cheng A.S., Lee C.W., Wong V.W., Sung J.J., Yu J. (2010). PPARgamma is essential for protection against nonalcoholic steatohepatitis. Gene Ther..

[115] Luo W., Xu Q., Wang Q., Wu H., Hua J. (2017). Effect of modulation of PPAR-γ activity on Kupffer cells M1/M2 polarization in the development of non-alcoholic fatty liver disease. Sci. Rep..

[116] Goyal O., Nohria S., Goyal P., Kaur J., Sharma S., Sood A., Chhina R.S. (2020). Saroglitazar in patients with non-alcoholic fatty liver disease and diabetic dyslipidemia: A prospective, observational, real world study. Sci. Rep..

[117] Krishnappa M., Patil K., Parmar K., Trivedi P., Mody N., Shah C., Faldu K., Maroo S., Parmar D. (2020). Effect of saroglitazar 2 mg and 4 mg on glycemic control, lipid profile and cardiovascular disease risk in patients with type 2 diabetes mellitus: A 56-week, randomized, double blind, phase 3 study (PRESS XII study). Cardiovasc. Diabetol..

[118] Boyer-Diaz Z., Aristu-Zabalza P., Andrés-Rozas M., Robert C., Ortega-Ribera M., Fernández-Iglesias A., Broqua P., Junien J.L., Wettstein G., Bosch J. (2021). Pan-PPAR agonist lanifibranor improves portal hypertension and hepatic fibrosis in experimental advanced chronic liver disease. J. Hepatol..

[119] Liu W.Y., Xie D.M., Zhu G.Q., Huang G.Q., Lin Y.Q., Wang L.R., Shi K.Q., Hu B., Braddock M., Chen Y.P. (2015). Targeting fibroblast growth factor 19 in liver disease: A potential biomarker and therapeutic target. Expert Opin. Ther. Targets.

[120] Roberts S.K., Majeed A. (2021). A short report on NGM282/aldafermin for the treatment of nonalcoholic steatohepatitis (NASH). Expert Opin. Ther. Targets.

[121] Harrison S.A., Rinella M.E., Abdelmalek M.F., Trotter J.F., Paredes A.H., Arnold H.L., Kugelmas M., Bashir M.R., Jaros M.J., Ling L. (2018). NGM282 for treatment of non-alcoholic steatohepatitis: A multicentre, randomised, double-blind, placebo-controlled, phase 2 trial. Lancet.

[122] Fisher F.M., Maratos-Flier E. (2016). Understanding the Physiology of FGF21. Annu. Rev. Physiol..

[123] Babaknejad N., Nayeri H., Hemmati R., Bahrami S., Esmaillzadeh A. (2018). An Overview of FGF19 and FGF21: The Therapeutic Role in the Treatment of the Metabolic Disorders and Obesity. Horm. Metab. Res. Horm.-Und Stoffwechs. Horm. Metab..

[124] Kliewer S.A., Mangelsdorf D.J. (2019). A Dozen Years of Discovery: Insights into the Physiology and Pharmacology of FGF21. Cell Metab.

[125] Jimenez V., Jambrina C., Casana E., Sacristan V., Muñoz S., Darriba S., Rodó J., Mallol C., Garcia M., León X. (2018). FGF21 gene therapy as treatment for obesity and insulin resistance. EMBO Mol. Med..

[126] Verzijl C.R.C., Van De Peppel I.P., Struik D., Jonker J.W. (2020). Pegbelfermin (BMS-986036): An investigational PEGylated fibroblast growth factor 21 analogue for the treatment of nonalcoholic steatohepatitis. Expert Opin. Investig. Drugs.

[127] Sanyal A., Charles E.D., Neuschwander-Tetri B.A., Loomba R., Harrison S.A., Abdelmalek M.F., Lawitz E.J., Halegoua-DeMarzio D., Kundu S., Noviello S. (2019). Pegbelfermin (BMS-986036), a PEGylated fibroblast growth factor 21 analogue, in patients with non-alcoholic steatohepatitis: A randomised, double-blind, placebo-controlled, phase 2a trial. Lancet.

[128] Kaufman A., Abuqayyas L., Denney W.S., Tillman E.J., Rolph T. (2020). AKR-001, an Fc-FGF21 Analog, Showed Sustained Pharmacodynamic Effects on Insulin Sensitivity and Lipid Metabolism in Type 2 Diabetes Patients. Cell Rep. Med..

[129] Seki E., De Minicis S., Gwak G.Y., Kluwe J., Inokuchi S., Bursill C.A., Llovet J.M., Brenner D.A., Schwabe R.F. (2009). CCR1 and CCR5 promote hepatic fibrosis in mice. J. Clin. Investig..

[130] Saiman Y., Friedman S.L. (2012). The role of chemokines in acute liver injury. Front. Physiol..

[131] Ortiz Zacarías N.V., van Veldhoven J.P.D., den Hollander L.S., Dogan B., Openy J., Hsiao Y.Y., Lenselink E.B., Heitman L.H., AP I.J. (2019). Synthesis and Pharmacological Evaluation of Triazolopyrimidinone Derivatives as Noncompetitive, Intracellular Antagonists for CC Chemokine Receptors 2 and 5. J. Med. Chem..

[132] Carter P.H., Brown G.D., Cherney R.J., Batt D.G., Chen J., Clark C.M., Cvijic M.E., Duncia J.V., Ko S.S., Mandlekar S. (2015). Discovery of a Potent and Orally Bioavailable Dual Antagonist of CC Chemokine Receptors 2 and 5. ACS Med. Chem. Lett..

[133] Barreyro F.J., Holod S., Finocchietto P.V., Camino A.M., Aquino J.B., Avagnina A., Carreras M.C., Poderoso J.J., Gores G.J. (2015). The pan-caspase inhibitor Emricasan (IDN-6556) decreases liver injury and fibrosis in a murine model of non-alcoholic steatohepatitis. Liver Int..

[134] Shiffman M., Freilich B., Vuppalanchi R., Watt K., Chan J.L., Spada A., Hagerty D.T., Schiff E. (2019). Randomised clinical trial: Emricasan versus placebo significantly decreases ALT and caspase 3/7 activation in subjects with non-alcoholic fatty liver disease. Aliment. Pharmacol. Ther..

[135] Bral M., Pawlick R., Marfil-Garza B., Dadheech N., Hefler J., Thiesen A., Shapiro A.M.J. (2019). Pan-caspase inhibitor F573 mitigates liver ischemia reperfusion injury in a murine model. PLoS ONE.

[136] Pejnovic N., Jeftic I., Jovicic N., Arsenijevic N., Lukic M.L. (2016). Galectin-3 and IL-33/ST2 axis roles and interplay in diet-induced steatohepatitis. World J. Gastroenterol..

[137] Iacobini C., Menini S., Ricci C., Blasetti Fantauzzi C., Scipioni A., Salvi L., Cordone S., Delucchi F., Serino M., Federici M. (2011). Galectin-3 ablation protects mice from diet-induced NASH: A major scavenging role for galectin-3 in liver. J. Hepatol..

[138] Weiskirchen R. (2015). Hepatoprotective and Anti-fibrotic Agents: It’s Time to Take the Next Step. Front. Pharm..

[139] Rotman Y., Sanyal A.J. (2017). Current and upcoming pharmacotherapy for non-alcoholic fatty liver disease. Gut.

[140] Tarry-Adkins J.L., Grant I.D., Ozanne S.E., Reynolds R.M., Aiken C.E. (2021). Efficacy and Side Effect Profile of Different Formulations of Metformin: A Systematic Review and Meta-Analysis. Diabetes Ther. Res. Treat. Educ. Diabetes Relat. Disord..

[141] Mehta A., Marso S.P., Neeland I.J. (2017). Liraglutide for weight management: A critical review of the evidence. Obes. Sci. Pract..

[142] Pose E., Trebicka J., Mookerjee R.P., Angeli P., Gines P. (2019). Statins: Old drugs as new therapy for liver diseases?. J. Hepatol..

[143] Schwabl P., Hambruch E., Budas G.R., Supper P., Burnet M., Liles J.T., Birkel M., Brusilovskaya K., Konigshofer P., Peck-Radosavljevic M. (2021). The Non-Steroidal FXR Agonist Cilofexor Improves Portal Hypertension and Reduces Hepatic Fibrosis in a Rat NASH Model. Biomedicines.

[144] Sinakos E., Liava C., Loomba R. (2022). Emerging advances in the pharmacologic treatment of nonalcoholic steatohepatitis and related cirrhosis. Ann. Gastroenterol..

[145] Chen M.M., Cai J.J., Yu Y., She Z.G., Li H. (2019). Current and Emerging Approaches for Nonalcoholic Steatohepatitis Treatment. Gene Expr..

[146] Polyzos S.A., Katsiki N. (2022). Semaglutide, cilofexor, and firsocostat for nonalcoholic steatohepatitis: A dance that may need more than one dancer. Hormones.

[147] Jiang L., Xiao D., Li Y., Dai S., Qu L., Chen X., Guo M., Wei H., Chen Y. (2021). Structural basis of tropifexor as a potent and selective agonist of farnesoid X receptor. Biochem. Biophys. Res. Commun..

[148] Schattenberg J.M., Pares A., Kowdley K.V., Heneghan M.A., Caldwell S., Pratt D., Bonder A., Hirschfield G.M., Levy C., Vierling J. (2021). A randomized placebo-controlled trial of elafibranor in patients with primary biliary cholangitis and incomplete response to UDCA. J. Hepatol..

[149] Sven M.F., Pierre B., Manal F.A., Quentin M.A., Elisabetta B., Vlad R., Philippe H.M., Bruno S., Jean-Louis J., Pierre B. (2020). A randomised, double-blind, placebo-controlled, multi-centre, dose-range, proof-of-concept, 24-week treatment study of lanifibranor in adult subjects with non-alcoholic steatohepatitis: Design of the NATIVE study. Contemp. Clin. Trials.

[150] Mayo M.J., Wigg A.J., Leggett B.A., Arnold H., Thompson A.J., Weltman M., Carey E.J., Muir A.J., Ling L., Rossi S.J. (2018). NGM282 for Treatment of Patients With Primary Biliary Cholangitis: A Multicenter, Randomized, Double-Blind, Placebo-Controlled Trial. Hepatol. Commun..

[151] Shao W., Jin T. (2022). Hepatic hormone FGF21 and its analogues in clinical trials. Chronic Dis. Transl. Med..

[152] Attia S.L., Softic S., Mouzaki M. (2021). Evolving Role for Pharmacotherapy in NAFLD/NASH. Clin. Transl. Sci..

[153] Zhou J., Huang N., Guo Y., Cui S., Ge C., He Q., Pan X., Wang G., Wang H., Hao H. (2019). Combined obeticholic acid and apoptosis inhibitor treatment alleviates liver fibrosis. Acta Pharm. Sinica. B.

[154] Harrison S.A., Marri S.R., Chalasani N., Kohli R., Aronstein W., Thompson G.A., Irish W., Miles M.V., Xanthakos S.A., Lawitz E. (2016). Randomised clinical study: GR-MD-02, a galectin-3 inhibitor, vs. placebo in patients having non-alcoholic steatohepatitis with advanced fibrosis. Aliment. Pharmacol. Ther..

[155] Cehic M.G., Muir C.A., Greenfield J.R., Hayward C., Jabbour A., Keogh A., Kotlyar E., Muthiah K., Macdonald P.S. (2019). Efficacy and Safety of Empagliflozin in the Management of Diabetes Mellitus in Heart Transplant Recipients. Transplant. Direct.

[156] Hsiang J.C., Wong V.W. (2020). SGLT2 Inhibitors in Liver Patients. Clin. Gastroenterol. Hepatol..

[157] Tacelli M., Celsa C., Magro B., Giannetti A., Pennisi G., Spatola F., Petta S. (2018). Antidiabetic Drugs in NAFLD: The Accomplishment of Two Goals at Once?. Pharmaceuticals.

[158] Lian J., Fu J. (2021). Pioglitazone for NAFLD Patients With Prediabetes or Type 2 Diabetes Mellitus: A Meta-Analysis. Front. Endocrinol..

[159] Tilinca M.C., Tiuca R.A., Niculas C., Varga A., Tilea I. (2021). Future perspectives in diabesity treatment: Semaglutide, a glucagon-like peptide 1 receptor agonist (Review). Exp. Ther. Med..

[160] Dibba P., Li A.A., Perumpail B.J., John N., Sallam S., Shah N.D., Kwong W., Cholankeril G., Kim D., Ahmed A. (2018). Emerging Therapeutic Targets and Experimental Drugs for the Treatment of NAFLD. Diseases.

[161] Yu Y., Cai J., She Z., Li H. (2019). Insights into the Epidemiology, Pathogenesis, and Therapeutics of Nonalcoholic Fatty Liver Diseases. Adv. Sci..

[162] Gomez-Dominguez E., Gisbert J.P., Moreno-Monteagudo J.A., Garcia-Buey L., Moreno-Otero R. (2006). A pilot study of atorvastatin treatment in dyslipemid, non-alcoholic fatty liver patients. Aliment. Pharmacol. Ther..

[163] Jun B.G., Cheon G.J. (2019). The utility of ezetimibe therapy in nonalcoholic fatty liver disease. Korean J. Intern. Med..

[164] Shen B., Lu L.G. (2021). Efficacy and safety of drugs for nonalcoholic steatohepatitis. J. Dig. Dis..

[165] Brodosi L., Marchignoli F., Petroni M.L., Marchesini G. (2016). NASH: A glance at the landscape of pharmacological treatment. Ann. Hepatol..

[166] Boner G., Cooper M.E., McCarroll K., Brenner B.M., de Zeeuw D., Kowey P.R., Shahinfar S., Dickson T., Crow R.S., Parving H.H. (2005). Adverse effects of left ventricular hypertrophy in the reduction of endpoints in NIDDM with the angiotensin II antagonist losartan (RENAAL) study. Diabetologia.

[167] Mancia G., Schumacher H. (2012). Incidence of adverse events with telmisartan compared with ACE inhibitors: Evidence from a pooled analysis of clinical trials. Patient Prefer. Adherence.

[168] Lake J.E., Tseng C.H., Currier J.S. (2013). A pilot study of telmisartan for visceral adiposity in HIV infection: The metabolic abnormalities, telmisartan, and HIV infection (MATH) trial. PLoS ONE.

[169] Peng C., Stewart A.G., Woodman O.L., Ritchie R.H., Qin C.X. (2020). Non-Alcoholic Steatohepatitis: A Review of Its Mechanism, Models and Medical Treatments. Front. Pharmacol..

[170] Ganguli S., DeLeeuw P., Satapathy S.K. (2019). A Review Of Current And Upcoming Treatment Modalities In Non-Alcoholic Fatty Liver Disease And Non-Alcoholic Steatohepatitis. Hepatic Med. Evid. Res..

[171] Roeb E. (2022). Diagnostic and Therapy of Nonalcoholic Fatty Liver Disease: A Narrative Review. Visc. Med..

[172] Pydyn N., Miekus K., Jura J., Kotlinowski J. (2020). New therapeutic strategies in nonalcoholic fatty liver disease: A focus on promising drugs for nonalcoholic steatohepatitis. Pharmacol. Rep..

